# Very few sites can reshape the inferred phylogenetic tree

**DOI:** 10.7717/peerj.8865

**Published:** 2020-07-08

**Authors:** Warren R. Francis, Donald E. Canfield

**Affiliations:** Department of Biology, University of Southern Denmark, Odense, Denmark

**Keywords:** Ctenophora, Porifera, Bias, Phylogenetics, Supermatrix

## Abstract

The history of animal evolution, and the relative placement of extant animal phyla in this history is, in principle, testable from phylogenies derived from molecular sequence data. Though datasets have increased in size and quality in the past years, the contribution of individual genes (and ultimately amino acid sites) to the final phylogeny is unequal across genes. Here we demonstrate that removing a small fraction of sites strongly favoring one topology can produce a highly-supported tree of an alternate topology. We explore this approach using a dataset for animal phylogeny, and create a highly-supported tree with a monophyletic group of sponges and ctenophores, a topology not usually recovered. Because of the high sensitivity of such an analysis to gene selection, and because most gene sets are neither standardized nor representative of the entire genome, researchers should be diligent about making intermediate analyses available with their phylogenetic studies. Effort is needed to ensure these datasets are maximally informative, by ensuring all genes are systematically sampled across relevant species. From there, it could be determined whether any gene or gene sets introduce bias, and then deal with those biases appropriately.

## Introduction

It has been over a decade since Rokas, Krüger & Carroll (2005) noted substantial challenges in reconciling the molecular phylogeny of metazoans, particularly with respect to deep nodes. In an early attempt to apply molecular sequence data to bilaterian evolutionary relationships, Dunn et al. (2008) had the surprising finding that ctenophores (comb jellies) emerged as the sister-group to the rest of metazoans (hereafter called Ctenophora-sister), contrary to the classically-held view that sponges were sister-group to all other animals (the hypothesis called Porifera-sister). A number of papers followed arguing both for and against each of these topologies (Philippe et al., 2009; Ryan et al., 2013; Whelan et al., 2015; Pisani et al., 2015; Simion et al., 2017; Whelan et al., 2017). Thus, despite over a decade of work, the deep branches of the animal tree remain inconclusive.

Relationships among non-bilaterian animals historically placed sponges as sister-group to remaining animals, which agreed with a scheme of “complexity” coming from the Aristotelian chain-of-being; sponges are simple animals, and therefore should be placed at the root of the animal tree. Although by this logic, the morphologically simplest animals, placozoans, might be considered the sister-group to all other animals. Ctenophores, on the other hand, had historically been placed in a group with cnidarians, called “coelenterata” or “radiata”, though detailed morphological analyses argued that every proposed synapomorphy of “coelenterata” was either uninformative (absence characters) or incorrect (Harbison, 1985), indicating they were falsely united.

Molecular phylogenetics is not without troubles as well, as the choice of genes used in phylogenetic reconstruction may have a substantial effect on the final tree. Shen, Hittinger & Rokas (2017) have shown that for many controversial nodes, some genes have very strong phylogenetic signals while other genes contain essentially none. This indicates a very high sensitivity to the genes that are included in the final dataset, and even what sites are included from those genes. While Shen, Hittinger & Rokas (2017) made some suggestions about how to resolve recalcitrant nodes, their method highlighted a potential risk of “stacking the deck” and generating a biased tree topology by selecting a set of genes that skew towards one topology.

Here we explicitly remove sites that strongly favor either Ctenophora-sister or Porifera-sister from a publicly available dataset, and then build a phylogenetic tree from the remaining sites. We demonstrate that with the removal of only 1.7% of sites, we can generate a tree with an alternate topology of metazoan phylogeny. We then discuss how sitewise filtering strategies could introduce substantial biases.

## Methods

### Datasets and processing

We re-analylzed dataset 16 from Whelan et al. (2015), the same dataset used in the re-analysis by Pisani et al. (2015) and by Shen, Hittinger & Rokas (2017) (hereafter called D16). This dataset was a filtered version of the main dataset used by Whelan et al. (2015), wherein genes and taxa with high long-branch scores were removed, and from that, the slowest-evolving half of the genes were analyzed.

**Figure 1:**
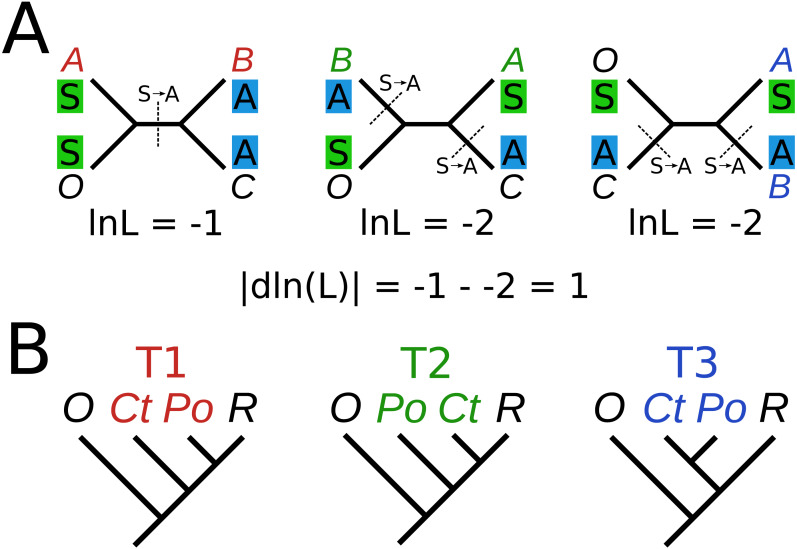
**Schematic of analysis.** (A) Three fixed trees differ by the position of groups A and B, relative to group C and outgroup O. Sites in the alignment either show 1 or 2 substitutions, depending on which tree is used. The substitutions do not have direction in time-reversible models, so the transition applies in either direction across the dotted lines. In this hypothetical example, the dln(L) between the maximum (-1) and median (-2) would be 1, indicating a strong site favoring T1. In this case, while T1 has the maximum likelihood, it is also the most parsimonious. (B) Concretely, in our study, T1 was the Ctenophora-sister hypothesis, T2 was the Porifera-sister hypothesis, and T3 was the paranimalia hypothesis. ‘Ct’ and ‘Po’ indicate ctenophores and sponges, respectively, while ‘O’ indicates non-metazoan outgroups (other opisthokonts) and ‘R’ indicates the rest of animals.

Sitewise likelihood calculations were generated using the method of Shen, Hittinger & Rokas (2017), with one difference. Briefly, this is a four-branch resolution problem, whereby the method takes three fixed trees and analyzes the likelihood at each site given the three possibilities (Fig. 1). Using the program RAxML, this is done with the option -f G. The likelihood values for each site for each tree are then directly compared, where the least negative means the most likely. However, in Shen, Hittinger & Rokas (2017), the strength of the site for each topology (dlnL) was calculated as the average of the absolute value of the three differences. Such approach would overestimate the strength of sites where one topology was substantially weaker (i.e., less likely) than the other two. Thus, we defined the strength of a site as the values of the maximum likelihood topology minus the score of the second best topology. Here “strong sites” are defined as sites where the absolute difference in log likelihood is greater than or equal to 0.5, the same threshold used by Shen et al. The vast majority of sites have differences in likelihood values that are close to zero (appx. 98% of sites, see Fig. S1), thus a dlnL score of 0.5 represents roughly 3 standard deviations above the mean.

To generate our experimental dataset (called the “weak” dataset), we started with the sitewise likelihood scores from Shen, Hittinger & Rokas (2017) for dataset D16 of Whelan et al. (2015), which were reformatted to a tabular file using a Python script sitewise_ll_to_columns.py. This was then used as the input for another script sitewise_get_strong_sites_2tree.py that calculated strong sites based on the first two trees, Ctenophora-sister and Porifera-sister, and removed sites with dlnL greater or equal to 0.5 that favored either of the two topologies, but not those supporting the third topology, the monophyly of sponges and ctenophores. This procedure removed 414 sites out of the total 23676 sites, only 1.7% (for comparison, human and zebrafish are 14% different across the entire alignment of this dataset). These scripts can be found at the gitHub repository: https://github.com/wrf/pdbcolor/tree/master/sitewise_scripts (commit tag: R1).

### Phylogenetics

We generated phylogenetic trees using RAxML v8.2.11 (Stamatakis, 2014) using the PROTGAMMALGF model and 100 bootstrap replicates with the “rapid boostrap” option (-f a). The same dataset was run in a Bayesian framework with Phylobayes-MPI v1.8 using the CAT-GTR model (Lartillot, Brinkmann & Philippe, 2007). Two chains were run in parallel for 2000 cycles and otherwise default parameters. Trees and run data can be found at the online repository: https://bitbucket.org/wrf/paranimalia-sites (commit tag: R2).

### Comparison across datasets

We compared the extent of alignment trimming and sitewise coverage across several phylogenetic datasets from previously published studies (see Table 1). For calculation of the trimmed fraction for each protein, we used the length of the alignment (excluding gaps) relative to the human reference protein from SwissProt. Because human proteins were not included in the Philippe 2009 or Ryan 2013 EST datasets, the human orthologs needed to be identified for each gene.

**Table 1:** Phylogenomic data sources: “Human proteins” column refers to the number of genes where the human ortholog was included in the final dataset. Numbers in brackets refer to the number of human orthologs recovered using our hmmsearch approach (see Methods). Total kept percent after trimming refers to the percent of sites that remained in the final dataset compared to the sum of the lengths of all untrimmed human orthologs.

Dataset name	Genes	Taxa	Sites	Average coverage (by gene)	Average coverage (by site)	Human proteins	Total kept percent after trimming	Reference
Philippe 2009	128	55	30257	82%	73.1%	0 (128)	86.0%	Philippe et al. (2009)
Ryan 2013 EST	406	70	88384	50	41.6	0 (396)	62.5	Ryan et al. (2013)
Whelan 2015 D1	251	76	81006	75	59.6	248	56.6	Whelan et al. (2015)
Borowiec 2015	1080	36	384981	87	75.8	1056	64.7	Borowiec et al. (2015)
Cannon 2016	212	78	44896	80	69.0	212	46.1	Cannon et al. (2016)
Simion 2017	1719	97	401632	74	60.7	1499	40.0	Simion et al. (2017)

To identify human orthologs in the Philippe 2009 and Ryan 2013 datasets, we developed a pipeline to retrieve genes from an existing alignment in additional species, called add_taxa_to_align.py. This pipeline makes use of hmmbuild and hmmsearch from the HMMER package v3.1b2 (Eddy, 2011) and the alignment program MAFFT v7.313 (Katoh, Rozewicki & Yamada, 2017). Briefly, for each gene in a supermatrix, a hidden Markov model is generated using hmmbuild, and this is used as the query for hmmsearch to search within a file of proteins from the new species. The results are filtered by multiple heuristics (cutoffs for e-value and bits/length are determined uniquely for each gene, and very short fragments are ignored), and the best sequence is added to the existing alignment using MAFFT, with the –addlong option. As the sole purpose of doing so was to assess what proportion of the original protein was used in the final alignment, we did not expect any problems due to mis-alignments of the new genes to the existing ones.

This script and related instructions are available at the gitHub repository: https://github.com/wrf/supermatrix.

### Robinson-Foulds distance

Pair-wise Robinson-Foulds distances were calculated using RAxML v8.2.11 (Stamatakis, 2014) using the option (-f r). Seven trees were used (see Table 2), including the three base trees used to calculate the site-wise likelihoods (from Shen, Hittinger & Rokas (2017)), the two trees of the current study from RAxML and phylobayes, as well as the original trees from Whelan et al. (2015) for datasets 16 and 10.

**Table 2:** Robinson-Foulds distances between trees used in this study.

Tree	T1	T2	T3	RAxML	Phylobayes	Whelan D16
T1 (Ctenophora-sister)	=	–	–	–	–	-
T2 (Porifera-sister)	2	=	–	–	–	-
T3 (Paranimalia)	2	2	=	–	–	-
RAxML tree (this study)	2	2	0	=	–	-
Phylobayes tree (this study)	6	6	4	4	=	-
Whelan 2015 D16 Tree	0	2	2	2	6	=
Whelan 2015 D10 Tree	6	8	8	8	12	6

## Results and Discussion

### Paranimalia is recovered regardless of model

**Figure 2:**
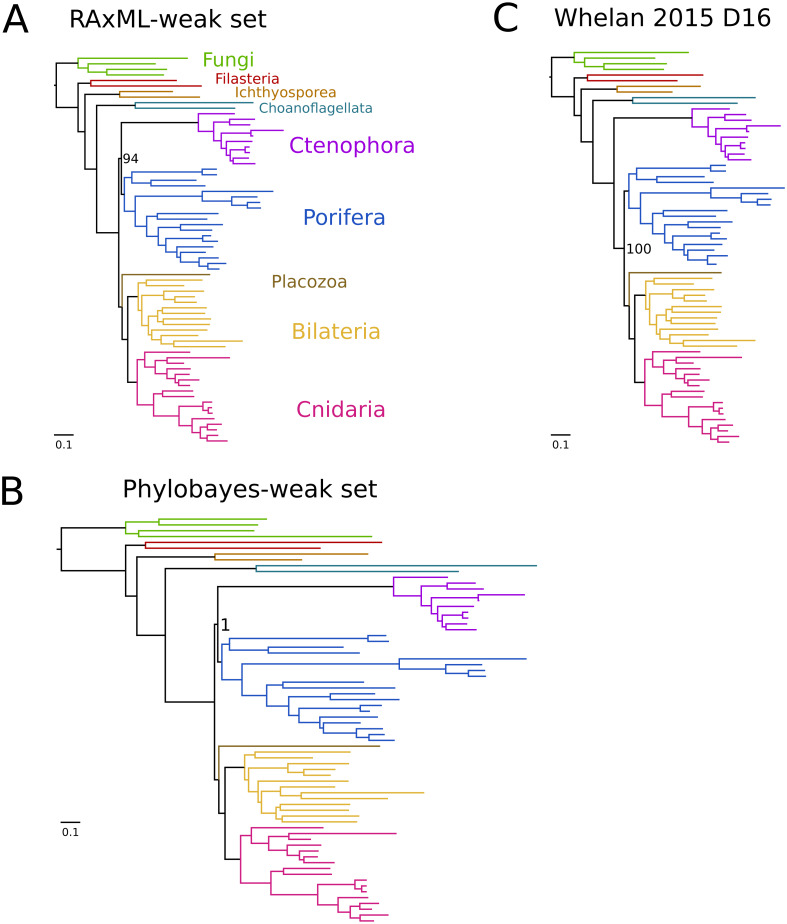
**Overview of phylogenetic trees.** (A) Tree from RAxML. (B) Tree from Phylobayes with CAT-GTR. Note the scale bars are the same but the phylobayes tree is substantially longer, likely due to increased substitutions predicted from the CAT model. (C) Original tree from Whelan et al. (2015) dataset 16, showing that, other than ctenophores and sponges, nearly all bipartitions are exactly the same before and after our processing. Most support values are removed for clarity.

By removing the “strong” sites from the supermatrix alignment (see Fig. 1 and Methods), we then generated two phylogenetic trees using two programs, RAxML (using the model PROTGAMMALGF) and phylobayes (under the model CAT-GTR) to assess the impact of these sites on the final tree. While trees from D16 had previously supported Ctenophora-sister (Whelan et al., 2015) or Porifera-sister (Pisani et al., 2015) under different conditions, both trees from our “weak” dataset strongly supported monophyly of ctenophores and sponges, (bootstrap:94; PP:1.0; Fig. 2), hereafter called “paranimalia”. This confirms that the sites removed contained the majority of phylogenetic information in support of Ctenophora-sister or Porifera-sister because neither of these topologies are recovered but the remainder of the tree is mostly identical to the D16 tree.

It had been suggested that the Ctenophora-sister topology occurred due to long-branch attraction artifact to distant outgroups (Philippe et al., 2009; Pisani et al., 2015), but we instead recovered “paranimalia” even with distant outgroups. Our results therefore indicate that influence of outgroups (i.e., long-branch attraction artifacts) is weaker than the intrinsic signal in the sites that favor “paranimalia”. Another possibility could be that the very sites that support Ctenophora-sister are those subject to the proposed “long branch attraction”.

### Few topological differences are found

**Figure 3:**
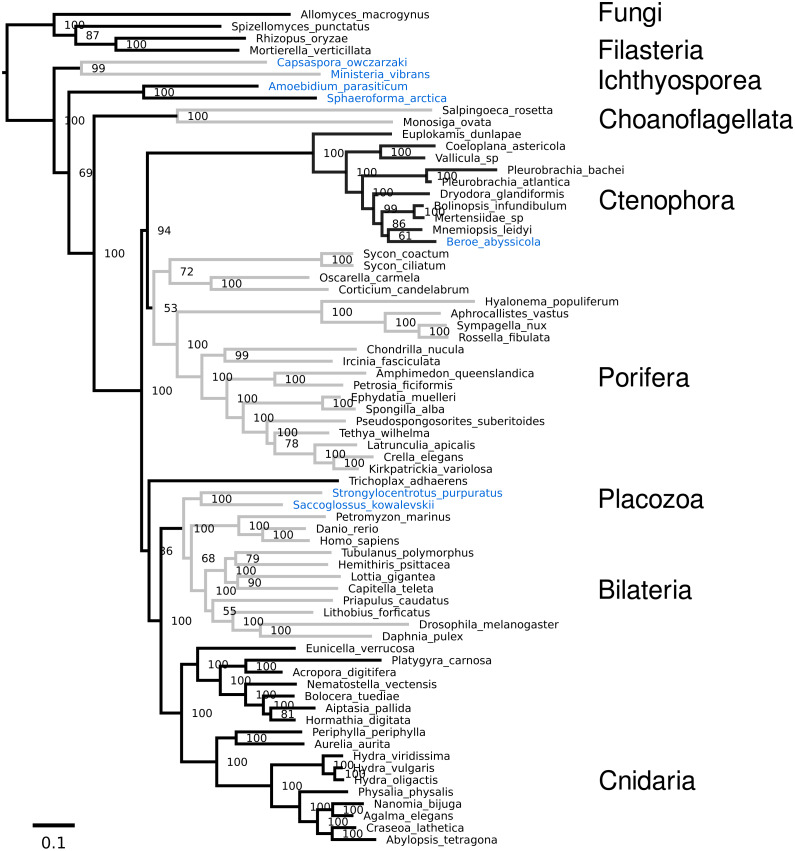
**RAxML tree:** Tree of the weak dataset from RAxML using the PROTGAMMALGF model. Taxa highlighted in red are moved relative to the original dataset 16 by Whelan et al. (2015). Taxa highlighted in blue are moved relative to dataset 10 from the same study.

**Figure 4:**
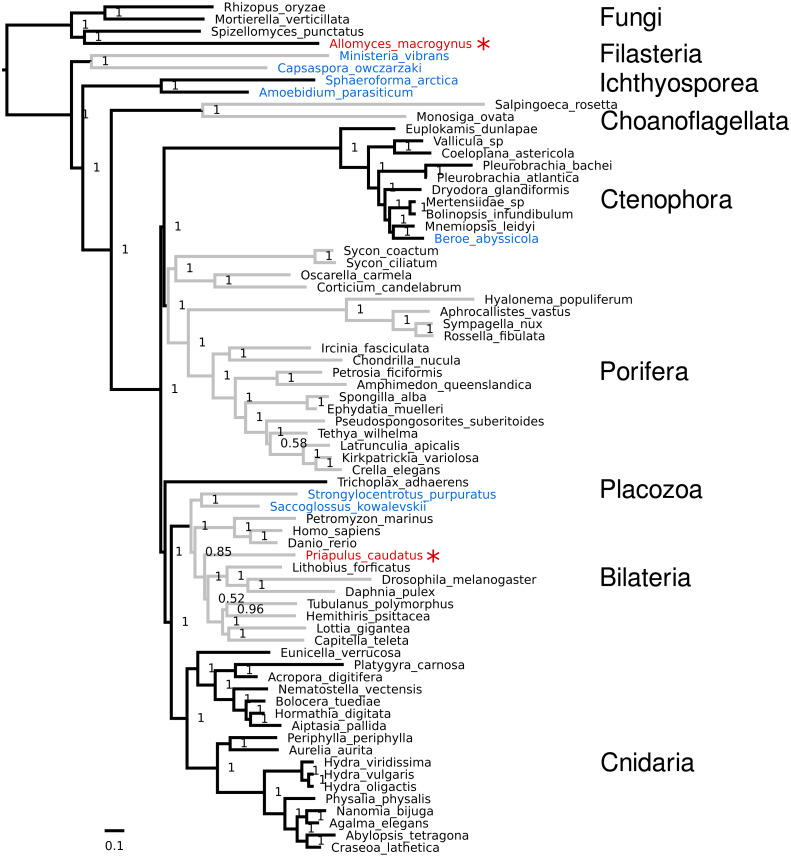
**Phylobayes tree.** Tree of the weak dataset from phylobayes using the CAT-GTR model. Taxa highlighted in red are moved relative to the original dataset 16 by Whelan et al. (2015). Taxa highlighted in blue are moved relative to dataset 10 from the same study.

The internal topology of nearly all phyla remains the same relative to the original dataset (Figs. 3 and 4), despite changing the position of the nodes for ctenophora and porifera (Table 2). This suggests that sites providing information for each bipartition are mostly independent. One obvious inconsistency is the placement of Ichthyosporea and Filasterea relative to dataset 10 by Whelan et al. (2015), as these two groups are swapped (see both Figs. 3 and 4).

For both analyses, Ambulacraria was recovered as sister to Bilaterians (71 bootstrap, PP 0.85), indicating paraphyletic deuterostomes. This topology was also recovered by Whelan et al. (2015) with their dataset 16, but not with dataset 10, which was used for the main figure in their publication (i.e., Figure 3 in Whelan et al. (2015)). This position of Ambulacraria was also found by Simion et al. (2017) after substantial trimming of the dataset, whereupon 70% of “heteropecillious” sites were removed (Roure & Philippe, 2011). Cannon et al. (2016) found this tree position occupied by the Xenacoelamorpha; this group includes the genus *Xenoturbella*, which was often recovered as sister to Ambulacraria within Deuterostomes (Philippe et al., 2009; Philippe et al., 2011). The recovery of Ambulacraria as sister to Bilaterians may indicate a relationship between heteropecilly (lineage-specific changes in the substitution process) and strong sites in a maximum likelihood framework. In other words, lineage-specific changes in proteins may be a major source of phylogenetic information, which may be problematic as the models assume a uniform substitution process across taxa.

Other small differences are evident (taxa highlighted blue in Figs. 3 and 4), such as the placement of the ctenophore *Beroe abyssicola* relative to *Mnemiopsis leidyi* (PP:1). Another difference is the placement of *Priapulus caudatus* as sister to protostomes, instead of just arthropods (PP:0.52). For the phylobayes tree, the RF distance plot (see supplement) shows that some changes still occur throughout the run, which is the repeated swapping of *Tethya wilhelma* and *Pseudospongosorites suberitoides*. However, both of these species are missing roughly half of the genes in the original dataset, which may explain the problems of convergence in this part of the tree.

### Most datasets are heavily trimmed

**Figure 5:**
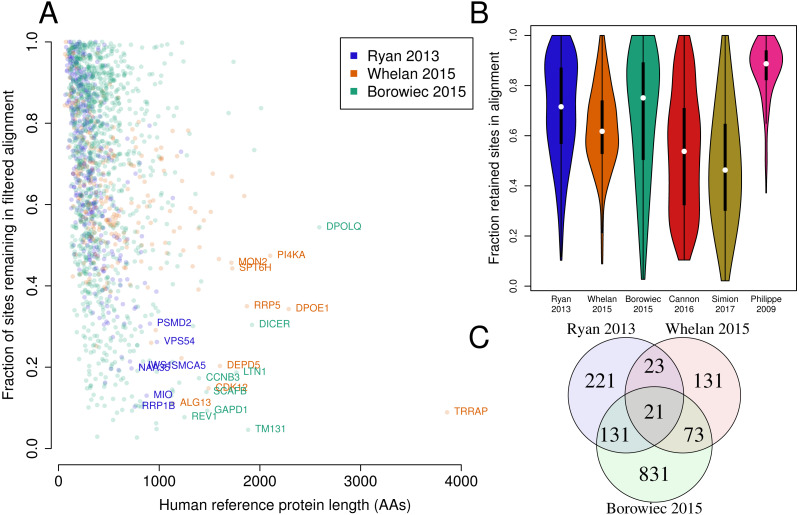
**Fraction of the original protein in the final alignment.** (A) Fraction of the original human protein that was used in the final alignment, meaning after any trimming steps, for the three metazoan datasets used by Shen, Hittinger & Rokas (2017), that is, the datasets from Ryan et al. (2013), Whelan et al. (2015), and Borowiec et al. (2015). Certain large and highly-trimmed protein names (Uniprot IDs) are indicated. (B) Violin plots showing the distribution of the kept fractions of the original proteins across the six datasets. Width is proportional to the normalized number of proteins with the coverage on the Y-axis. Simion 2017 was the least retained after trimming (average of 48%) while Philippe 2009 was the most (average of 86%). (C) Venn diagram of the proteins used in each study, out of 1,431 total proteins across all three studies.

In our weak dataset, only 1.7% of sites have been removed. The original dataset (D16) had already been trimmed by an average of 30% per protein, compared to the reference proteins. While such trimming strategies make sense for regions that cannot be aligned, one study found that nearly all programs will overtrim, resulting in an overall less-supported phylogeny than if no trimming were done at all (Tan et al., 2015). Even across the six published studies in Fig. 5 that we compare, none of them include an unfiltered version for analysis, so the effect of these removed sites or domains is unknown.

As there is typically no examination of which sites are filtered, it is easy to imagine incidentally removing sites that favor a particular hypothesis, as we had specifically done in this study. The most trimmed study, (Simion et al., 2017) had removed over half of the amino acids of each protein on average compared to the human reference proteins. That study began with almost 1 million amino acids of total native proteins to an alignment of just over 400k amino acids (Table 1). As sites affecting deep nodes may account for only a fraction of 1% of all sites, exclusion of 60% of the original sites may substantially affect the resulting inferences. We note, however, that this does not indicate that any given filtering strategy will not necessarily affect the phylogenetic inference in a particular direction. As noted above, all of the studies that we examined make use of alignment trimming, but none of them provided the untrimmed datasets so the effects of trimming are unknown.

### Difficulty in reconciling different studies

There are almost an infinite number of possible hypotheses of metazoan phylogeny, but most of these are unlikely, and thus we concern ourselves with a limited set of competing hypotheses of animal phylogeny, namely Ctenophora-sister and Porifera-sister. The “paranimalia” hypothesis was only generated here as an example, but the two phyla (Porifera and Ctenophora) share some qualities, such as the absence of the HIF oxygen sensing pathway (Mills et al., 2018).

Many phylogenies remain controversial because differences in gene set (Nosenko et al., 2013), substitution model (Feuda et al., 2017), taxon sampling, and missing data (Roure, Baurain & Philippe, 2013) have profound effects on the inferred tree. Although these factors influence a phylogenetic inference in test datasets, most of these issues cannot be adjusted or fixed in existing datasets. For instance, although the dataset by Ryan et al. (2013) had only 50% occupancy, it may be practically infeasible to recover the missing genes and demonstrate that these have an effect. Generating new data does not directly solve this problem; across the three studies (Whelan et al. (2015), Borowiec et al. (2015), and Ryan et al. (2013)), there are a total of 1,431 genes, yet despite similar pipelines for identifying genes (and mostly the same species), only 21 genes are common between the three studies. Thus, more recent studies are not replacements for older ones, as they are still inferring the phylogeny from a different set of genes from different species, each providing a different perspective of evolution.

Other technical factors like introduction of newer versions of software make it difficult to compare between datasets and results. A proper comparison of different pipelines or analysis strategies may require downloading or re-assembling the source data, finding orthologous genes across all species, filtering paralogs or incomplete transcripts, aligning, trimming, and finally generating the tree, i.e., completely redoing the entire study. It must further be stressed that there is substantial debate of how to go about every one of these steps.

### Limitations of sitewise analysis

Shen, Hittinger & Rokas (2017) had analysed the contributions of individual sites against trees with a fixed topology to discern which sites favor each tree. Such a method is highly sensitive to taxon sampling, and likelihood scores can be calculated even in cases where it appears to be inappropriate, or makes little biological sense. For instance, in the Borowiec et al. (2015) dataset, there was only one ctenophore (*Mnemiopsis leidyi*) and one sponge (*Amphimedon queenslandica*), yet strong sites favoring Ctenophora-sister or Porifera-sister were still calculated even when the gene being analyzed was absent for one or both of the two species. Potentially, genes that were absent in ctenophore or sponge species should have been excluded. Therefore, in order for the results to be meaningful, essentially all sites should be occupied for all relevant taxa, in this case meaning all ctenophores, sponges, and outgroups.

There is a semantic problem in how the results are described. It is common to say there is “robust” support for a hypothesis in phylogenetics based purely on the bootstrap or posterior probability, but these two values do not reflect the fraction of sites favoring the two hypotheses of interest. Even considering the results of Shen, Hittinger & Rokas (2017) at face value, the only datasets that have more than 1 taxon per phylum for ctenophores and sponges for most genes are the Whelan et al. (2015) datasets. Of these, barely above 50% of all sites (both strong and weak sites) favor Ctenophora-sister. Shen, Hittinger & Rokas (2017) argued this was still sufficient support for the Ctenophora-sister hypothesis. However, sitewise likelihood values are calculated for all sites, including constant sites and sites with neither ctenophores nor sponges. As weak sites are essentially phylogenetic noise, it would be more accurate to say this hypothesis is *slightly* or *marginally* favored, while 98% of sites do not affect this part of the tree. Thus, there is not “robust” support for either topology. The resolution of the deep nodes of the tree, regardless of method or model, is extremely poor, and the statistical strategies to validate the approach (bootstrapping or posterior probability) do not reflect the true uncertainty of the data. Given the tenuous support for any of the deep nodes of animal phylogeny, it seems reasonable to say that we simply lack the information to resolve this, and should, at this time, defer to the null hypothesis that this node is still unresolved until better models are implemented (Feuda et al., 2017) or alternate strategies to infer phylogeny are applied (Ryan et al., 2013; Pisani et al., 2015).

### Other examination of bias in datasets

The work by Feuda et al. (2017) had attempted to examine the effects of strong sites as a function of substitution model. However, the “outlier-excluded” dataset used in their re-analysis was produced by removing outliers without considering the topology they favored and this site-selection methodology actually resulted in a dataset depleted in Ctenophora-sister favoring sites (all of the seven outliers favored Ctenophora-sister). A tree supporting Porifera-sister should therefore be expected from the analysis of this dataset as genes strongly supporting Ctenophora-sister were removed, but not those favoring Porifera-sister or any other systematic bias. Our results indicated that removal of sites favoring a specific topology (in our case, both Ctenophora-sister and Porifera-sister) can produce a highly supported tree favoring another topology for which sites were not removed (i.e., paranimalia).

### Do existing filtering strategies create a bias?

Many filtering strategies may have negative effects on the inferred tree (Tan et al., 2015). Whether this specifically applies to datasets in our study cannot be determined here, as none of the studies examined here provided a pre-trimmed dataset. We therefore could not examine a causal relationship between “normal” filtering, and differences in the inferred phylogeny, though this was not our objective. Despite this, our results indicate that removal of a small fraction of sites (under 2%) can dramatically change the phylogenetic inference, and ultimately the hypotheses of evolution. Many studies trim at least 40% of sites from the reference proteins (Fig. 5B), with some proteins trimmed by over 90%, potentially being reduced to a single domain (Fig. 5A). As strong sites are not evenly distributed among or within genes, it is conceivable that trimming of a single domain could remove multiple strong sites for one hypothesis or another, though not consistently for any given protein or dataset. This would naturally affect the final tree, though the direction of such effect could not be predicted from our data.

### What would make an unbiased set?

As shown by Shen, Hittinger & Rokas (2017), some genes have disproportionate signal in favor of one phylogenetic topology. This in itself is not a bias. This merely indicates that the phylogenetic inference is highly sensitive to gene and site selection. That is to say, genes without strong sites are not unbiased, they are merely uninformative for the node in question, just as genes with many strong sites are not biased either. The presence of strong sites could become a bias if some other processing strategy were to favor inclusion of those genes over others. It seems unlikely that unbiased genes could be selected *a priori*, meaning that selection of any given gene (e.g., 18S or CO1) or set of genes (ribosomal or mitochondrial) for phylogenetics is likely to introduce some kind of stochastic effect.

Compared to the current practice of selecting genes for phylogenetics, there is only one way to have an unbiased set, whether deliberately or algorithmically. This would require the inclusion all proteins, including those with multiple copies. Because of the difficulty in resolving species trees from multi-copy gene trees, algorithmic improvements are probably necessary. This may also demand that all species used in phylogenetic reconstructions have sequenced genomes to ensure that all genes are sampled, as bona fide gene losses cannot be identified with transcriptomes. From a set of all genes, it could then be possible to examine whether such biases exist, and what genes may present an unbiased perspective of evolution according to our current models.

## Conclusions

Using the example of metazoan phylogeny, we have demonstrated that minor manipulations to large datasets may affect the final phylogenetic tree, and ultimately the evolutionary interpretations. As noted by Shen, Hittinger & Rokas (2017), given that a few genes may drive the phylogeny of highly dynamic or controversial regions of the tree of life, *we should be skeptical of analyses that consider any controversial phylogeny to be a solved problem*. As our results have shown, the metazoan tree is not solved, and current practices in phylogenetics remove large amounts of data, which can easily affect the final phylogeny in presently-unknown ways. It is apparent that phylogenetic relationships that have been historically controversial cannot simply be resolved by additional data. The tape of life has clearly played out in only one way, but whether we can reconstruct each frame with the technology and data presently available is uncertain.

##  Supplemental Information

10.7717/peerj.8865/supp-1 Supplemental Information 1 Supplemental analyses

## Additional Information and Declarations

### Data Availability

The following information was supplied regarding data availability: Input data and results are available at:
https://bitbucket.org/wrf/paranimalia-sites.
