# Supplementary material for: Very few sites can reshape the inferred phylogenetic tree
Source: PeerJ. 2020 Jul 8;8:e8865. doi: 10.7717/peerj.8865 (PMC7353913; doi:10.7717/peerj.8865)
Supplement: Supplemental Information 1 [file peerj-08-8865-s001.pdf]

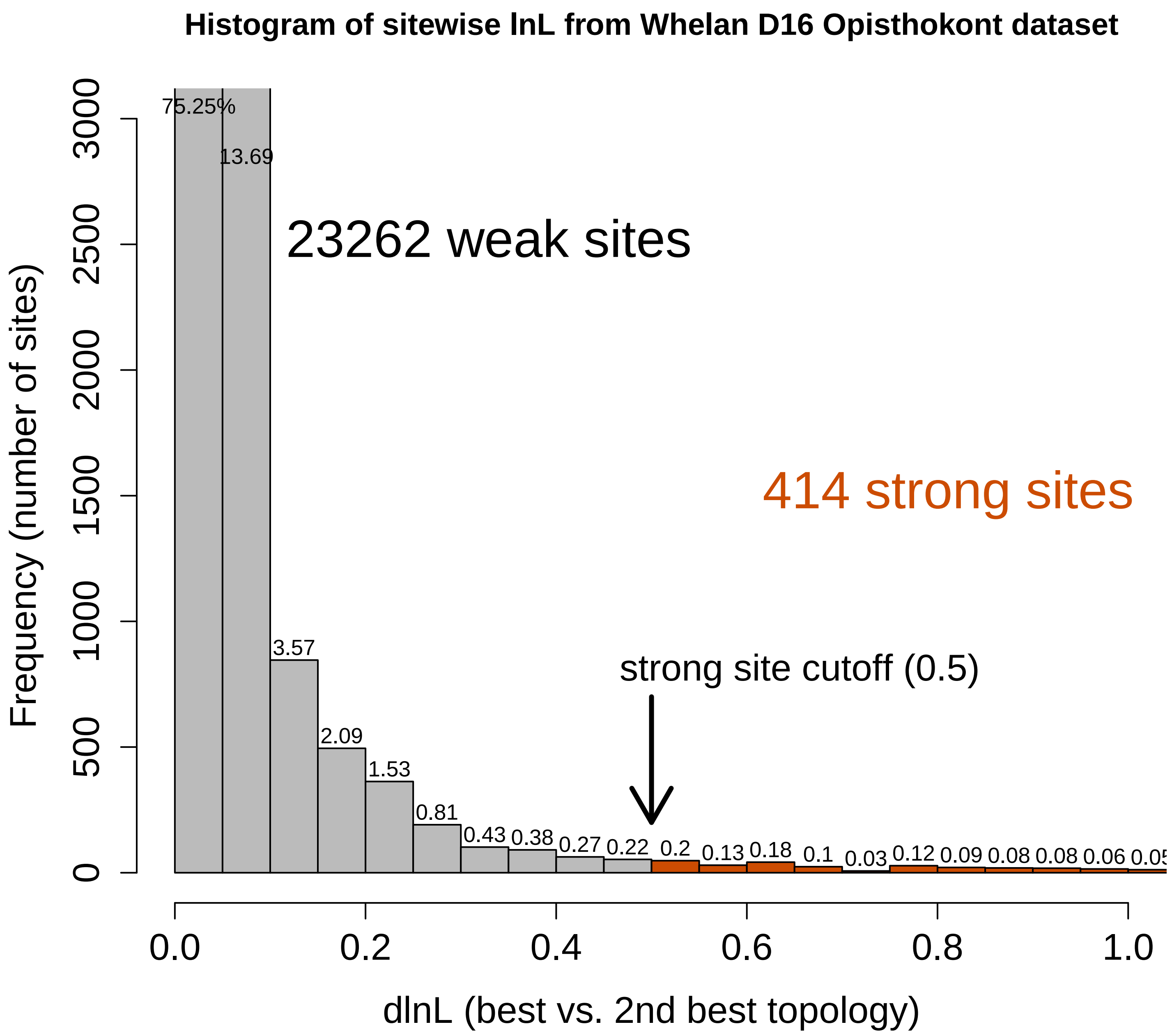
Histogram of sitewise lnL from Whelan D16 Opisthokont dataset
Frequency (number of sites)
0
500
1000
1500
2000
2500
3000
75.25%
13.69
3.57
2.09
1.53
0.81
0.43
0.38
0.27
0.22
0.2
0.13
0.18
0.1
0.03
0.12
0.09
0.08
0.08
0.06
23262 weak sites
414 strong sites
strong site cutoff (0.5)
0.0
0.2
0.4
0.6
0.8
1.0
dlnL (best vs. 2nd best topology)

RAxML_perSiteLLs.Whelan_D16_Opisthokonta_gene_lk.tab

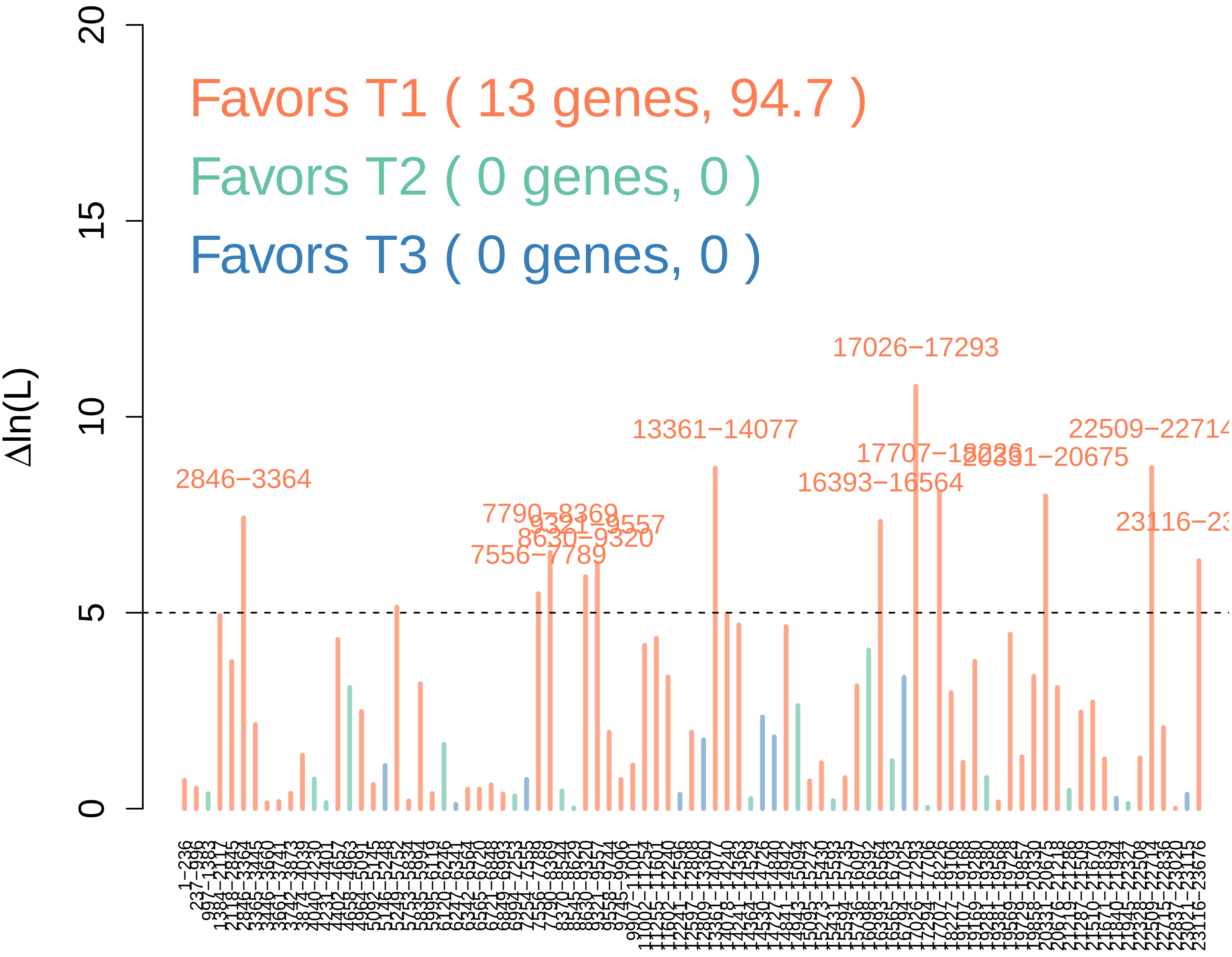

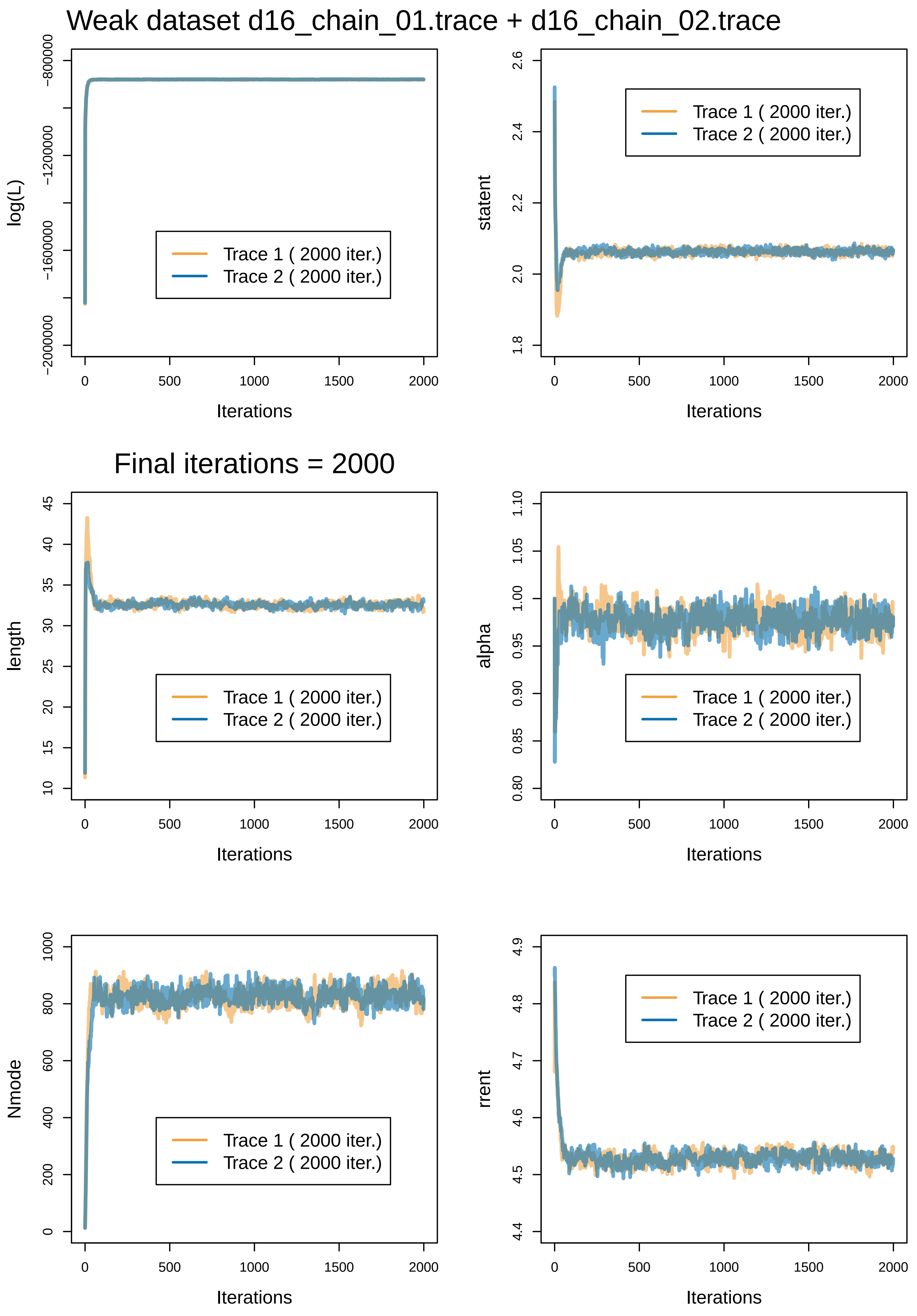
Weak dataset d16_chain_01.trace + d16_chain_02.trace
Final iterations = 2000
log(L)
statent
length
alpha
Nmode
rrent
Iterations
Trace 1 (2000 iter.)
Trace 2 (2000 iter.)

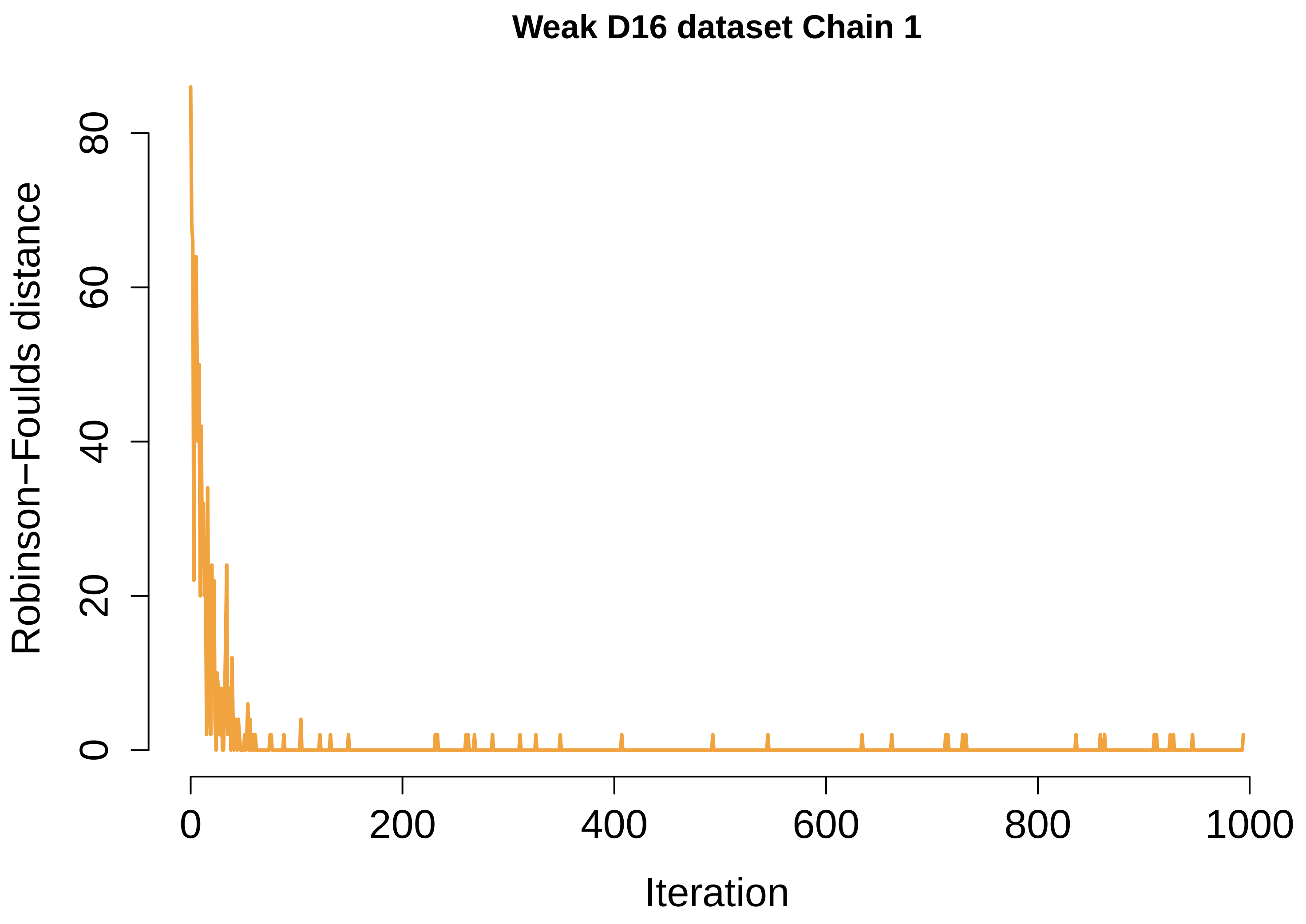
Weak D16 dataset Chain 1
Robinson−Foulds distance
0
20
40
60
80
0
200
400
600
800
1000
Iteration

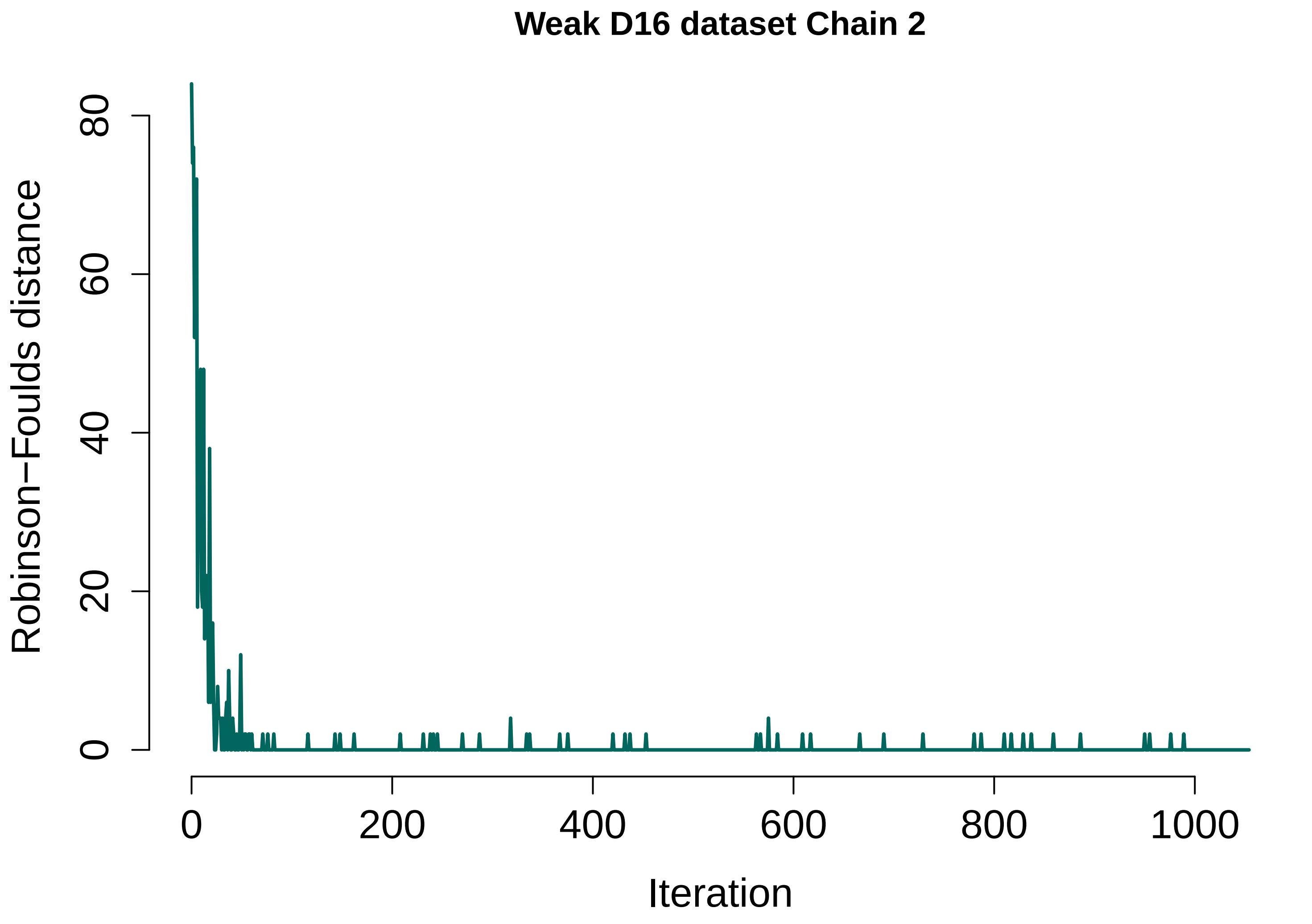
Weak D16 dataset Chain 2
Robinson−Foulds distance
0
20
40
60
80
0
200
400
600
800
1000
Iteration
